# Dietary Behaviors Associated With Fruit and Vegetable Consumption, Marion County, Indiana, 2005

**Published:** 2011-04-15

**Authors:** Karl W. Staser, Robert M. Saywell, Terrell W. Zollinger, Srujana Kunapareddy, P. Joseph, Virginia A. Caine

**Affiliations:** Department of Family Medicine and the Bowen Research Center, Indiana University School of Medicine, Indianapolis, Indiana; Indiana University, Bowen Research Center; Department of Family Medicine and the Bowen Research Center, Indiana University School of Medicine, Indianapolis, Indiana; Department of Family Medicine and the Bowen Research Center, Indiana University School of Medicine, Indianapolis, Indiana; Health and Hospital Corporation of Marion County, Marion County Health Department, Indianapolis, Indiana; Health and Hospital Corporation of Marion County, Marion County Health Department, Indianapolis, Indiana

## Abstract

**Introduction:**

Eating inadequate amounts of fruits and vegetables is associated with diminished health, and most Americans fall short of the Centers for Disease Control and Prevention's recommendation to eat at least 2 servings of fruit and 3 servings of vegetables each day. This study assessed behaviors associated with fruit and vegetable consumption in adults.

**Methods:**

A cross-sectional, random-digit–dialed telephone survey of 4,784 adults living in Marion County (Indianapolis), Indiana, measured demographic characteristics, personal health data, food consumption, food label use, and other eating habits. Multivariate logistic regressions were used to assess the association between selected dietary behaviors and fruit and vegetable consumption, controlling for demographic characteristics.

**Results:**

Behaviors associated with adequate versus inadequate consumption of fruits and vegetables were frequent snacking on healthy foods (odds ratio [OR], 2.54), eating meals at home (OR, 2.09), using nutrition labels when making purchases (OR, 1.52), and using "heart healthy" symbols and other food information labels when ordering from restaurants (OR, 1.41). Frequent red meat consumption was negatively associated with adequate consumption of fruits and vegetables (OR, 0.64).

**Conclusions:**

Healthful snacking, food label use, and eating meals prepared at home may improve dietary quality. Our measure of adequacy may also be useful in future studies assessing dietary behavior and diet composition.

## Introduction

Low levels of fruit and vegetable consumption are associated with diminished health, including increased risk for obesity (1), heart disease and stroke (2,3), type 2 diabetes (4), and some types of cancer (5). Accordingly, federal and state government programs and websites such as www.fruitsandveggiesmatter.gov (6) and INShape Indiana (7) promote increased fruit and vegetable consumption. Despite these efforts, most Americans fall short of the Centers for Disease Control and Prevention's "5 A Day" recommendation to eat at least 2 servings of fruit and 3 servings of vegetables each day. Two-thirds of adults in the United States eat fewer than 2 servings of fruit per day and nearly three-quarters eat fewer than 3 servings of vegetables per day (8).

Interventions intended to increase fruit and vegetable consumption would benefit from increased awareness of the factors that are associated with greater fruit and vegetable consumption. To varying degrees, studies have characterized the association between healthful diets or fruit and vegetable consumption and demographic factors, including sex, race/ethnicity, age, education, income, geographic area, body mass index (BMI), and smoking status (9,10). Several studies have examined the association of dietary behaviors, such as television viewing while eating, food sources (eg, supermarket availability, restaurants, home preparation), nutrition label use, and snacking with healthful diets and increased fruit and vegetable consumption (11). Most of the behavioral studies have focused on subpopulations, including adolescents, low-income families, women, Hispanics, or African Americans (9,12). Among the broad-based studies, Carlson and Gerrior (10) sought to examine the dietary behaviors associated with dietary quality, focusing mainly on the effect of food source and availability. However, we are not aware of any broad-based study specifically assessing multiple dietary behaviors associated with adequate consumption of fruits and vegetables.

The objective of this study was to examine the adequacy of fruit and vegetable consumption as it relates to dietary behavior using a large cross-sectional survey. Specifically, this study analyzes the association of fruit and vegetable consumption with red meat and soda consumption, snacking behaviors, the setting and frequency of meals, and the use of food labels, controlling for demographic characteristics, smoking status, and BMI. This study additionally proposes expanded use of the Healthy Eating Index (HEI) to delineate categorical variables for large-scale association studies.

## Methods

The Marion County Health Department and the Bowen Research Center of Indiana University developed a cross-sectional, population-based telephone survey to gather baseline data on the prevalence of obesity, physical activity patterns, and health indicators among English-speaking and Spanish-speaking adults (aged 18 or older) residing in Marion County, Indiana. The protocol for this study received approval in February 2005 from the Indiana University-Purdue University Indianapolis IUPUI and Clarian Institutional Review Board (study number 0502-56). Each interview lasted about 18 minutes. We intentionally oversampled Hispanics, blacks, and Hispanic and black men to assure sufficient responses for race/ethnicity and sex-specific analyses, and we weighted responses to ensure that race/ethnicity and sex results matched the demographic makeup of Marion County. The Survey Research Center at IUPUI conducted the random-digit–dialed telephone survey from February through June 2005 by using trained interviewers and computer-assisted telephone interview software (WinCati, Sawtooth Technologies, Inc, Northbrook, Illinois). Of 14,675 eligible sampled households, 32.6% completed the survey, as calculated by the American Association for Public Opinion Research's response rate definition number 4 to calculate the survey response rate (13). Of the 4,784 completed surveys, 4,165 participants provided the necessary data to be included in the analysis. We excluded all participants who did not provide the required data on age, sex, race/ethnicity, educational attainment, height and weight, and food intake.

We developed the survey from the 2004 Behavioral Risk Factor Surveillance System questionnaire, the National Health and Nutrition Examination Survey 2001-2002, and the International Physical Activity Questionnaire (14-16). We added some original questions, especially those pertinent to the local community, and health department personnel pilot-tested several versions of the questionnaire. Demographic data gathered were age, sex, race/ethnicity, educational attainment, smoking status, and height and weight (to calculate BMI); other data gathered covered physical activity, food intake, and eating patterns. We excluded the few (n = 96) survey respondents who indicated that they were of a racial group other than non-Hispanic white, non-Hispanic black, or Hispanic, and we excluded the 56 respondents who did not answer the race item on the questionnaire. We computed BMI by multiplying the participant's self-reported weight in pounds by 703 and dividing by the participant's height in inches squared, yielding kg/m^2^ (17).

Survey participants estimated their daily servings of fruits and vegetables. For fruit, we described a serving as a medium-sized fruit, a half-cup of chopped fruit, or a quarter cup of dried fruit. For vegetables, we described a serving as a cup of raw vegetables or a half cup of cooked vegetables. Participants also estimated their daily intake of fruit and vegetable juices, in 6-ounce servings. We collected consumption data on the following items: daily servings of red meat, described as "the size of a deck of cards"; daily servings of nondiet 12-ounce sodas; and daily servings of "less healthy foods, such as chips, a piece of pie or cake, cookies, candy, or ice cream."

### Other dietary behavior data assessed

Participants reported how often they snacked between meals (number of times per typical week) and how often that snack was "a healthy food such as fresh fruit, vegetables, or nuts." They further reported the setting and frequency of meals (eaten at a restaurant, prepared at home, eaten in the car, eaten as a primary activity in the absence of television, computer, or other activities, and eaten while viewing television). We regrouped continuous variables into "frequent" and "infrequent" categories, on the basis of median value for the dietary behavior analyzed, for entry into the logistic regression analyses. Values equal to or above the median were "frequent" and values below the median were "infrequent." For example, for the dietary behavior "meals eaten at home," the median value of all included responses was 14. Thus, we defined "frequent" as a response of 14 or more times per week, and we defined "infrequent" as 13 or fewer.

Finally, participants reported their use of food labels or symbols (such as "heart symbols") on restaurant menus, their use of nutrition labels to determine what foods to buy from the market, and their use of food labels to determine serving size for meal preparation. We re-categorized response options "all of the time," "most of the time," "some of the time," "rarely," or "never" into "all or most of the time" or "some of the time or never" for the logistic regression analysis.

### Consumption grouping

We used the Harris-Benedict equation to compute each person's basal metabolic rate (BMR) by using self-reported height, weight, age, and sex data (18). In lieu of adequate exercise data, we multiplied the BMR by 1.5 to correspond to "moderate daily activity," yielding the estimated energy requirement (EER), as derived from guidelines at www.mypyramidtracker.gov (19). From the EER, we computed the number of cups of both fruits and vegetables that a participant would need to eat to receive the maximum score on the Healthy Eating Index 2005 (HEI-2005) for daily intake of fruits and vegetables, as outlined by the US Department of Agriculture (20,21). The HEI-2005 assigns a maximum score to a person who eats at least 0.8 cups of fruit per 1,000 calories eaten and 1.1 cups of vegetables per 1,000 calories eaten. For example, a person with a 2,000 calories per day EER would need to eat at least 1.6 cups of fruit and 2.2 cups of vegetables to receive a maximum score on the HEI-2005.

Using these data and the food consumption data, we grouped participants into 1 of 4 categories: *inadequate, fruit adequate only, vegetable adequate only,* and *adequate.* The *inadequate* group did not meet the HEI-2005 maximum score for either fruits or vegetables. The *fruit adequate only* group met the HEI-2005 maximum score for fruit but not for vegetables. The *vegetable adequate only* met the HEI-2005 maximum score for vegetables but not for fruit. The *adequate* group met the HEI-2005 maximum score for both fruit and vegetables.

### Statistical analysis

We performed 3 multivariate logistic analyses by using SAS version 9.1.3 (SAS Institute, Inc, Cary, North Carolina). The dependent variables for the 3 analyses were placement within the *adequate* category compared with *inadequate*, *fruit adequate only*, or *vegetable adequate only* categories. The demographic variables were controlled for in the analyses. The independent variables were the dietary behaviors. *P* values less than .05 were considered statistically significant.

## Results

Most respondents were non-Hispanic white (71.0%); 24.4% were non-Hispanic black and 4.6% were Hispanic. Half (52.1%) of the respondents were women. On the basis of computed BMI from self-reported height and weight data, more than half of the respondents (61.3%) were overweight (BMI of 25.0 kg/m^2^-29.9 kg/m^2^), obese (30.0 kg/m^2^-34.9 kg/m^2^), or severely obese (≥35.0 kg/m^2^). One quarter of the respondents were current smokers, and almost two-thirds of respondents reported attending "some college" or being a college graduate. The mean respondent age was 45.2 years.

Using the EER in conjunction with self-reported fruit and vegetable consumption and the parameters described in the HEI-2005, most respondents were categorized as *inadequate* consumers of fruits and vegetables (57.4%). The next largest group was *fruit adequate only* (22.7%). The *vegetable adequate only* (9.3%) and *adequate* groups (10.6%) had similar proportions.

The respondents reporting eating *adequate* fruits and vegetables had strikingly different demographic characteristics from the *inadequate* and *fruit adequate only* consumers: a higher proportion were women (74.6%), fewer smoked (15.2%), most had at least some college education (70.9%), a lower proportion had an elevated BMI (>25.0 kg/m^2^, 43.3%), and they were older (average age, 49.2 y) (Table 1). Non-Hispanic black participants were overrepresented in the *fruit adequate only* (32.9%) and *adequate* (28.9%) groups (compared with 24.4% of total respondents).

The behavior most strongly associated with placement in the *adequate* group versus the *inadequate* group was snacking on healthful foods such as fresh fruit, vegetables, or nuts all the time or most of the time (OR, 2.54), followed by frequent eating of meals prepared at home (OR, 2.09) (Table 2). Other significant behaviors associated with being placed in the *adequate* group versus the *inadequate* group included using nutrition labels all or most of the time when making purchasing decisions at the market and reading "heart healthy" symbols and other food information labels when ordering from restaurant menus all or most of the time. However, frequent red meat consumption was negatively associated with placement in the *adequate* group versus the *inadequate* group (OR, 0.64).

Being placed in the *adequate* group versus *fruit adequate only* group was also associated with snacking on healthful foods such as fresh fruit, vegetables, or nuts all the time or most of the time (OR, 1.74) (Table 3) and secondarily with frequently eating meals prepared at home. Frequently eating red meat was not a behavior associated with placement in the *adequate* versus *fruit adequate only* group. The behaviors associated with placement within the *adequate* group compared with the *inadequate* or the *fruit adequate only* groups were similar, but the differences were not significant for nutrition label use at the market and at restaurants between the *adequate* versus *fruit adequate only* group.

Two dietary behaviors were associated with placement in the *adequate* group versus *vegetable adequate only* group: snacking on healthful foods such as fresh fruit, vegetables, or nuts all the time or most of the time (OR, 1.65) and frequent eating of meals prepared at home (OR, 1.65) (Table 4). No other behavior was associated with *adequate* placement in this analysis.

## Discussion

We have used the HEI-2005 (20) to create fruit and vegetable "adequacy" categories to assess dietary behaviors associated with fruit and vegetable consumption while controlling for demographic variables, BMI, and smoking status. Although the HEI-2005 is principally used to assess diet composition and quality independent of total caloric consumption, the HEI-2005's designers and others have postulated expanded uses for the tool (20,21). Our categorical analyses have permitted a more stringent categorization of respondents' fruit and vegetable adequacy based on their estimated caloric intake. This approach may afford additional levels of power, especially in a large cross-sectional study, because the HEI-based categories provide more precision in placing respondents into the adequacy groupings compared with the more simplified approaches such as "5 A Day." Moreover, when we reanalyzed our results by using the simplified approach, respondents reporting "5 A Day" consumption, compared with our *adequate* consumption grouping, demonstrated increased average BMI, smoking rate, and other demographic variables associated with decreased scores on previously published HEI-based studies (8,22).

Thus, we surmise that using the HEI-2005 in conjunction with self-reported fruit and vegetable consumption and anthropometric data increases our power to detect behaviors likely to be associated with high-quality diets, including a sufficient proportion of fruits and vegetables. However, we stress caution in interpreting these results. First, the study relies on cross-sectional self-reported data. Ideally, we would use food diaries or 24-hour recall and standardized anthropometric data acquisition methods, although these methods would be difficult to execute in a large-scale survey. Moreover, detailed food frequency surveys may be suitable to rank or categorize people based on the amount of self-reported fruit and vegetable intake (23), especially in large-scale studies. Regardless, our methods subject all analyses to recall biases and skewed self-perceptions, such as a scenario in which a person who reports frequent healthful snacking simultaneously tends to overestimate fruit and vegetable consumption or underestimate anthropometric data. The literature has well established the confounding effect of differing self-perceptions among survey respondents, and the definition of a "meal" versus a "snack" could differ substantially among survey participants (24). Second, these data do not imply causality. Likewise, this study cannot rule out influences of unexplored factors (eg, exact geographic area, food source access, cultural differences of self-perception and reporting) for eating adequate amounts of fruits and vegetables.

Despite these limitations, the adequacy categories correlate to demographic variables in previously published "5 A Day" and HEI-based reports (8), granting external validity to our categorization method. Moreover, we found that the stringent consumption categorizations increased our ability to categorize food intake, allowing a more accurate assessment of the impact of behaviors associated with eating adequate amounts of fruits and vegetables while controlling for demographic variables. Although we cannot ascertain from our data set whether this method provides a more valid assessment of associated behaviors, these results could inform future study designs based on food diaries, 24-hour recall, or clinically acquired data. A future longitudinal study could perform in-depth analysis of whether these behaviors precede, accompany, or follow eating adequate amounts of fruits and vegetables. A similar study, either longitudinal or cross-sectional, could ask whether and to what extent differing self-perceptions of diet and lifestyle substantially affect both actual and reported eating of adequate amounts of fruits and vegetables.

Although our study identified frequent healthful snacking as a behavior associated with meeting our definition of eating adequate amounts of fruits and vegetables, it does not prove causality between "healthful snacking" and fruit and vegetable adequacy. Despite this, we note recent studies finding "healthful snacking" as an effective strategy for improving diet quality in multiple subpopulations (25). Thus, our data may tangentially support these studies.

Previous studies found an association between increased frequency of home-prepared meals and diet quality or improved health outcomes (26). Our data may indicate that meals eaten or prepared at home include more fruits and vegetables. Alternatively, persons who have access or desire to prepare and eat meals at home may either prefer fruits and vegetables or perceive themselves as eating fruits and vegetables.

Although many studies have implicated the consumption of red meat — and, perhaps more importantly, red meat high in saturated fat — with increased cancer risk (27), we are not aware of any studies directly assessing the association between red meat consumption and inadequate consumption of fruits and vegetables. A detailed analysis of this association with red meat consumption would require further study.

Food label use at restaurants and at the market was associated with placement in the *adequate* group versus the *inadequate* group. This association may reflect a correlation between diet quality and education about, concern with, or access to nutritional information. A laboratory-based study found that nutrition label availability directly decreased participants' total caloric consumption (28). Thus, efforts to educate people on food label use, such as SNAP-Ed, might directly promote diet quality, including increased fruit and vegetable consumption.

We found that dietary fruit and vegetable adequacy, as defined by using data from a self-reported cross-sectional study, was most strongly associated with healthful snacking and frequent eating of meals prepared at home. Other factors identified included frequent food label use and infrequent red meat consumption. Additionally, we demonstrated an expanded usage for the HEI-2005, whereby we categorized and analyzed single dietary components (ie, fruits and/or vegetable consumption) by using self-reported anthropometric and consumption data. Our data demonstrate that future investigations can use the HEI to uncover potential behavioral associations with facets of diet quality while controlling for demographic variables. These associations may serve to confirm or contrast previous interventional or longitudinal studies and to inform future studies assessing a particular behavior or demographic associated with dietary adequacy.

## Figures and Tables

**Table 1:** Comparison of Demographic Characteristics and Fruit and Vegetable Consumption Among Survey Participants, Marion County, Indiana, 2005^a^

**Characteristic**	Inadequate Fruit and Vegetable Consumers (n = 2,391)	Adequate Fruit and Inadequate Vegetable Consumers (n = 944)	Adequate Vegetable and Inadequate Fruit Consumers (n = 388)	Adequate Fruit and Vegetable Consumers (n = 442)	*P* Value^b^
**Sex, %**					
Men	56.9	46.4	25.9	25.4	<.01
Women	43.1	53.6	74.1	74.6	
**Race/ethnicity, %**					
Non-Hispanic white	75.7	61.5	86.0	67.5	<.01
Non-Hispanic black	20.9	32.9	12.7	28.9	
Hispanic	3.4	5.6	1.2	3.5	
**Smoking, %**					
No	71.7	76.7	74.5	84.8	<.01
Yes	28.3	23.3	25.5	15.2	
**Some college or more, %**					
No	37.3	40.2	27.4	29.1	<.01
Yes	62.7	59.8	72.6	70.9	
**Weight status,^c^ %**					
Underweight	0.6	1.7	4.2	2.9	<.01
Normal weight	31.2	45.5	43.6	53.8	
Overweight	36.6	32.5	34.0	30.7	
Obese	18.6	12.1	11.6	7.0	
Severely Obese	13.1	8.2	6.5	5.6	
**Age group, %**					
18-34 y	34.3	42.9	22.3	28.6	<.01
35-60 y	49.1	35.2	53.6	41.8	
>60 y	16.6	21.9	24.1	29.6	
**Average age, y**	44.5	43.9	49.1	49.2	NA

Abbreviation: NA, not applicable.

a Adequacy of fruit or vegetable consumption was calculated by using each person's basal metabolic rate (from self-reported data on height, weight, age, and sex) multiplied by 1.5 (corresponding to "moderate daily activity") to yield the estimated energy requirement (EER). From the EER, we computed the number of cups of fruits or vegetables that a participant would need to eat for the maximum score on the Healthy Eating Index-2005 (HEI-2005) (20,21). The group with *adequate* fruit and vegetable consumption met the HEI-2005 maximum score for both fruits and vegetables. The *fruit adequate only* group met the HEI-2005 maximum score for fruit but not for vegetables. The *vegetable adequate only* group met the HEI-2005 maximum score for vegetables but not for fruit. The *inadequate* group did not meet the HEI-2005 maximum score for either fruits or vegetables.

b *χ* ^2^ test.

c Body mass index of <18.5  kg/m^2^ was defined as underweight; 18.5  kg/m^2^-24.9 kg/m^2^, normal weight; 25.0  kg/m^2^-29.9  kg/m^2^, overweight; 30.0  kg/m^2^-34.9  kg/m^2^, obese; and ≥35.0 kg/m^2^, severely obese.

**Table 2:** Multiple Logistic Regression for *Adequate* Versus *Inadequate* Fruit and Vegetable Consumption, Controlling for Age, Sex, Race/Ethnicity, Educational Attainment, Smoking Status, and Body Mass Index, Among Survey Participants, Marion County, Indiana, 2005^a^

**Independent Variable**	Odds Ratio^b^ (95% CI)	*P* Value^c^
Frequent meals eaten in the car	1.09 (0.80-1.48)	.59
Frequent meals eaten that are home-prepared	2.09 (1.57-2.78)	<.01
Frequent meals eaten as a primary activity	1.25 (0.94-1.65)	.12
Frequent meals eaten at a restaurant	1.01 (0.76-1.35)	.94
Frequent meals eaten while watching television	1.05 (0.76-1.40)	.71
Frequent soft drink consumption	1.31 (0.94-1.84)	.11
Frequent snack consumption	1.09 (0.83-1.44)	.52
Frequent red meat consumption	0.64 (0.46-0.88)	<.01
Frequent food label use at the market	1.52 (1.11-2.08)	<.01
Frequent food label use to determine servings	1.21 (0.88-1.67)	.23
Frequent food label use at restaurants	1.41 (1.06-1.87)	.02
Frequent healthful snacking	2.54 (1.95-3.30)	<.01

Abbreviation: CI, confidence interval.

a Adequacy of fruit or vegetable consumption was calculated by using each person's basal metabolic rate (from self-reported height, weight, age, and sex data) multiplied by 1.5 (corresponding to "moderate daily activity") to yield the estimated energy requirement (EER). From the EER, we computed the number of cups of fruits or vegetables that a participant would need to eat for the maximum score on the Healthy Eating Index-2005 (HEI-2005) (20,21). The group with *adequate* fruit and vegetable consumption met the HEI-2005 maximum score for both fruits and vegetables. The *inadequate* group did not meet the HEI-2005 maximum score for either fruits or vegetables.

b The *inadequate* consumption group was the referent group for consumption. Frequency was dichotomized into high and low based on the median value among the participants surveyed; for each variable, the referent group was low frequency. Thus, the odds ratio relates to the likelihood of *adequate* consumption given high frequency of food type or behavior, controlling for the demographic characteristics listed.

c χ^2^ test.

**Table 3:** Multiple Logistic Regression for *Adequate* Versus *Fruit Adequate Only* Consumption, Controlling for Age, Sex, Race/Ethnicity, Educational Attainment, Smoking Status, and Body Mass Index, Among Survey Participants, Marion County, Indiana, 2005^a^

**Independent Variable**	Odds Ratio^b^ (95% CI)	*P* Value^c^
Frequent meals eaten in the car	1.25 (0.89-1.74)	.21
Frequent meals eaten that are home-prepared	1.55 (1.16-2.08)	<.01
Frequent meals eaten as a primary activity	0.91 (0.68-1.22)	.52
Frequent meals eaten at a restaurant	1.37 (0.99-1.89)	.06
Frequent meals eaten while watching television	1.21 (0.89-1.65)	.21
Frequent soft drink consumption	1.20 (0.84-1.72)	.31
Frequent snack consumption	0.85 (0.64-1.13)	.26
Frequent red meat consumption	0.65 (0.46-0.92)	.01
Frequent food label use at the market	1.28 (0.93-1.77)	.14
Frequent food label use to determine servings	1.22 (0.86-1.72)	.27
Frequent food label use at restaurants	1.33 (0.97-1.82)	.08
Frequent healthful snacking	1.74 (1.33-2.28)	<.01

Abbreviation: CI, confidence interval.

a Adequacy of fruit or vegetable consumption was calculated by using each person's basal metabolic rate (from self-reported height, weight, age, and sex data) multiplied by 1.5 (corresponding to "moderate daily activity") to yield the estimated energy requirement (EER). From the EER, we computed the number of cups of fruits or vegetables that a participant would need to eat for the maximum score on the Healthy Eating Index-2005 (HEI-2005) (20,21). The group with *adequate* fruit and vegetable consumption met the HEI-2005 maximum score for both fruits and vegetables. The *fruit adequate only* group met the HEI-2005 maximum score for fruit but not for vegetables.

b The *fruit adequate only* consumption group was the referent group for consumption. Frequency was dichotomized into high and low based on the median value among the participants surveyed; for each variable, the referent group was low frequency. Thus, the odds ratio relates to the likelihood of *adequate* consumption given high frequency of food type or behavior, controlling for the demographic characteristics listed.

c χ^2^ test.

**Table 4:** Multiple Logistic Regression for *Adequate* Versus *Vegetable Adequate Only* Consumption, Controlling for Age, Sex, Race/Ethnicity, Educational Attainment, Smoking Status, and Body Mass Index, Among Survey Participants, Marion County, Indiana, 2005^a^

**Independent Variable**	Odds Ratio^b^ (95% CI)	*P* Value^c^
Frequent meals eaten in the car	0.94 (0.64-1.38)	.74
Frequent meals eaten that are home-prepared	1.65 (1.16-2.36)	<.01
Frequent meals eaten as a primary activity	0.82 (0.58-1.17)	.26
Frequent meals eaten at a restaurant	0.93 (0.65-1.34)	.71
Frequent meals eaten while watching television	0.95 (0.66-1.35)	.76
Frequent soft drink consumption	1.55 (0.97-2.43)	.05
Frequent snack consumption	1.28 (0.92-1.76)	.14
Frequent red meat consumption	0.84 (0.56-1.26)	.40
Frequent food label use at the market	0.89 (0.59-1.34)	.57
Frequent food label use to determine servings	0.92 (0.64-1.34)	.66
Frequent food label use at restaurants	1.01 (0.70-1.46)	.97
Frequent healthful snacking	1.65 (1.20-2.27)	<.01

Abbreviation: CI, confidence interval.

a Adequacy of fruit or vegetable consumption was calculated by using each person's basal metabolic rate (from self-reported height, weight, age, and sex data) multiplied by 1.5 (corresponding to "moderate daily activity") to yield the estimated energy requirement (EER). From the EER, we computed the number of cups of fruits or vegetables that a participant would need to eat for the maximum score on the Healthy Eating Index-2005 (HEI-2005) (20,21). The group with *adequate* fruit and vegetable consumption met the HEI-2005 maximum score for both fruits and vegetables. The *vegetable adequate only* group met the HEI-2005 maximum score for vegetables but not for fruit.

b The *vegetable adequate only* consumption group was the referent group for consumption. Frequency was dichotomized into high and low based on the median value among the participants surveyed; for each variable, the referent group was low frequency. Thus, the odds ratio relates to the likelihood of *adequate* consumption given high frequency of food type or behavior, controlling for the demographic characteristics listed.

c χ^2^ test.
